# Crime-Specific Recidivism in Criminal Justice Clients with Substance Use—A Cohort Study

**DOI:** 10.3390/ijerph19137623

**Published:** 2022-06-22

**Authors:** Anna Karlsson, Anders Håkansson

**Affiliations:** 1 Department of Clinical Sciences Lund, Psychiatry, Faculty of Medicine, Lund University, 221 00 Lund, Sweden; anna.karlsson.5043@med.lu.se; 2 Malmö Addiction Center, Region Skåne, 205 02 Malmö, Sweden

**Keywords:** substance use disorder, criminal justice, recidivism, prison, criminal behavior

## Abstract

Criminal recidivism is a major global concern. There is a well-known association between substance use disorders and offending. Yet, little is known about crime-specific recidivism. The aim of this study was to investigate the relationship between specific substance use and crime-specific recidivism. The study is based on 4207 Swedish prison clients with substance use assessed with Addiction Severity Index interviews between 2001 and 2006. Clients were followed for an average of 2.7 years. Risk factors for criminal recidivism were assessed with the Cox regression analysis. Sixty-eight percent of the clients returned to the criminal justice system. Apart from well-known risk factors such as male gender and young age, amphetamine, injection drug use, prior prosecution for violent and property crime, as well as homelessness and psychiatric problems, were risk factors for criminal recidivism. Sedatives and cannabis were, in this setting, negative risk factors for general recidivism. Age, heroin and injection drug use elevated the risks of recidivism to property and drug crime. Alcohol was associated with violent recidivism. When analysing different categories of crime separately, risk factors differed substantially. This further highlights the need for crime-specific research. Identifying crime-specific risk factors should be an important part of improving rehabilitation into society after imprisonment and hopefully decrease recidivism.

## 1. Introduction

A large portion of criminal acts are committed by previously sentenced individuals. Unfortunately, few countries keep extensive statistics on criminal recidivism, making research in the field challenging. A five-year follow-up after release from prison indicated reconviction rates around 70% in the UK, 60% in France and 55% in the US. Although reconviction rates vary considerably across nations, criminal recidivism is a major global concern [1].

There is a well-known relationship between substance use disorders (SUD) and criminal offending [2,3,4]. A meta-analysis by Bennet et al., from 2008 concludes that the odds of offending are three- to four-times greater for people with SUD [2], and the prevalence of SUD is substantially higher in prisoners than in the general population [5,6]. Furthermore, drug-related criminality constitutes a large economic problem for society, and it has been argued that lowering alcohol and drug-related crime by 10% in the US would save society approximately USD 4.25 billion dollars [4].

Mental health disorders are common amongst people with SUD [7,8,9,10]. Some studies argue that psychiatric problems increase the risk of offending [10,11,12], yet others have shown the opposite [13,14]. A systematic review from 2002 by Fazel and Danesh shows that personality disorders are ten-times more common in a prison population and that depression and psychosis are over-represented [15]. There is also evidence that mental disorders in combination with SUD increase the risk of offending [16]. Yet, the importance of psychiatric comorbidity as a risk factor for criminality has been questioned [14]. It has been suggested that people suffering from serious mental illness alone have the same risk of reoffending as individuals with no SUD or psychiatric diagnosis [17].

It is known that male gender and young age are linked to recidivism and criminality in general. Poor living conditions and prior criminal behaviour have also been shown to predict reoffending. Although there is a substantial amount of previous research on substance use and general criminal recidivism, little is known about the role of separate types of substances and type of crime [2].

Regarding alcohol and crime, however, it has been established that alcohol increases the risk of violent behaviour and violent crime [3,18]. Both a pattern of regular drinking and acute influence of alcohol intake increase the risk of committing violent assaults [19].

In fact, variations in the activity of monoamine oxidase A (MAOA), an enzyme involved in the metabolism of several important neurotransmitters such as serotonin, noradrenalin, and dopamine, may, to some extent, explain inter-individual differences regarding the relationship between use of alcohol and violent behaviour [20]. Greater levels of alcohol consumption have also shown to increase the risk of such behaviour, although this might be the case only for those with the highly active MAOA [21].

Low executive cognitive functioning might also play an important role in understanding why certain individuals are more susceptible to becoming aggressive in the context of alcohol consumption [22]. Thus, the relationship between violent crime and alcohol is complex.

Regarding stimulants such as amphetamine and cocaine and violent crime, research is somewhat inconclusive [3]. There are studies that show a correlation between amphetamine and violent crime [11,23]. Other studies suggest that amphetamines do not increase aggressive behaviour [3,24], and might in fact decrease violent behaviour [25]. An Australian study stated that an increase in cocaine intake amongst people who inject drugs increased the risk of committing violent assaults [26]. Yet, research on cocaine administration has, thus far, not been able to demonstrate a direct link between cocaine intake and aggression [2]. Thus, further research on amphetamines, cocaine and crime is needed [2,3].

High doses of benzodiazepines have, in some studies, shown to increase the risk of violent offending, especially in previously violent individuals [18,27]. It seems as if benzodiazepines can act as both a stimulant and inhibitor of aggression, and that these contradictory effects might depend on dosage [3].

It has also been suggested that psychiatric comorbidity and SUD only slightly alter the risk of violent recidivism and that it would be of more value to concentrate on assessments of demographic and criminal history factors in order to predict violent reoffending [12].

Drug crime and property crime are two categories of crime with high rates of recidivism [28]. Property crime is common among drug users in order to finance drugs, especially amongst those with heavier and more expensive addictions such as heroin, and criminal recidivism is frequent [29]. A British study by Gossop et al., suggested that reductions in the level of heroin intake reduced the amounts of criminal acts, especially property crimes [29]. Injection drug use itself has also been stated as a risk factor for property crime and criminal recidivism in general [30].

Research on cannabis and crime have been somewhat inconclusive [2]. However, it is likely that cannabis elevates the risk of crime in general, solely due to the fact that it is illegal, and that cannabis use only increase risks of committing drug-related crime [31].

The aim of this study was to investigate the relationship between the use of specific substances and crime-specific recidivism. A hypothesis in the design of this study was that different risk factors, especially the use of different specific substances, may predict different patterns of criminal recidivism.

The following questions were addressed: (1) Are different risk factors associated with different patterns of criminal recidivism? (2) Which risk factors, including specific patterns of substance use, mediate criminal recidivism to crime in general and to violent, drug and property crime amongst prison clients with substance use disorders?

## 2. Materials and Methods

### 2.1. Material

This study is based on the Addiction Severity Index (ASI) database of the Swedish Prison and Probation Service. The ASI is a standardized and structured interview which aims to measure problems related to the usage of alcohol and illicit drugs [32]. The interview concerns areas of physical and mental health, the use of drugs and alcohol, employment, legal problems, family and other relations [32,33].

The ASI has been increasingly used within the Swedish Prison and Probation Service since 2001 for clients with known or suspected substance use problems [34]. The ASI version used, ASI-X, is a development of the European standard version, EuropASI [33]. The results of the ASI-X are used as a basis for the implementation of individualized care, and data are stored for future research [35]. Access to the ASI data requires permission from any of the six regional boards of ethical vetting in Sweden. This study was approved by the Ethics Committee of Lund, Sweden (reference number 2011/272). In accordance with the decision of the ethics committee, based on the retrospective register design and the fact that data are not identified, no informed consent from study participants was required. 

The instrument has been widely assessed and has an acceptable validity [32,36,37,38,39,40]. The present ASI material has been used in several other studies assessing different aspects of this population [10,41,42,43].

The interviews on which the present study relies were carried out between January 2001 and August 2006. For each client interviewed during this period, the Swedish Prison and Probation Service collected data on index and potential recidivist crime from the Swedish Criminal Records Registry between January 2001 and November 2010. The Swedish Prison and Probation Service also sent the personal identification number to the Swedish National Board of Health and Welfare, which supplied information on date of death for diseased individuals. This information was retrieved from the National Death Registry until 31 December 2008. Deaths hereafter were retrieved from the Criminal Records Registry. We were given the information without personal identification numbers. 

The material in total contains 7493 interviews with 7085 clients. Amongst these 5122 were interviewed in prison. We excluded 17 clients since they had been interviewed with a different interview form, the Adolescent Drug Abuse Diagnosis (ADAD). Hereafter, 26 clients were excluded because the interviewer had suspected that the client gave inadequate answers or misinterpreted the questions. In the present study, the aim was to include a cohort of individuals with manifest substance use-related problems. For this reason, and in the absence of diagnostic information at baseline from the prison setting, only individuals who reported any main drug were included, i.e., individuals who reported either alcohol, any of the illicit drugs included, a combination of alcohol and drugs, or a combination of different drugs, i.e., excluding individuals who reported to have “no primary drug” or for whom this information was missing. For this reason, 820 clients were excluded. Finally, no index crime was found for 52 of the clients between January 2001 and November 2010. The remaining number of clients, 4207, were included in the analyses.

### 2.2. Design

The index crime in this study was determined as the first criminal episode for which the client was sentenced during the time of this study (January 2001–August 2006). In cases where the index sentence contained several verdicts, the index crime was determined to be the main crime, i.e., the most serious offence. Recidivism was defined as any re-entry into the criminal justice system, both for any crime, and for whether they entered the criminal justice system for each of the specific crimes assessed. Apart from studying criminal recidivism in general, we chose to examine four categories of clients based on their index crime. Amongst the 4207 clients we selected the three largest categories of crime: drug crime (*n* = 1331, 32%), property crime (defined as any crime related to stealing, robbery, burglary, receiving of stolen goods and similar crimes, *n* = 1238, 29%) and violent crime (*n* = 710, 17%). Whenever more than one crime was included in the verdict, the main crime, i.e., the most serious offence, was included. 

When studying criminal recidivism we chose the same categories as for the index crime: all offences (*n* = 2888, 69%), drug offences (*n* = 890, 21%), property crime (*n* = 777, 18%) and violent crime (*n* = 277, 7%). Criminal recidivism was defined as any return to the criminal justice system, using data provided by the Criminal Records Registry. For each index, crime predictors for recidivism to all crime, to the same crime and to violent crime were studied.

Apart from the analyses of substance use, we included several risk factors which have been previously addressed in the literature [2,7,8,9,10,11,12,13,14]. The risk factors examined were the number of substances misused and misuse during one year of each of the substances alcohol, heroin, other opioids, sedatives, cocaine, amphetamine and cannabis, injection drug use during one year, prior episode of psychiatric inpatient treatment, psychiatric medication at any time, prior suicide attempts, homelessness 30 days prior to intake to the criminal justice system, age, male gender and self-reported prior prosecution for drug, violent and property crime.

### 2.3. Statistical Methods

Analyses were carried out using SPSS Statistics version 24 (IBM Corp., Armonk, NY, USA). All potential risk factors were first analysed in a Pearson Correlation Coefficient analysis. The highest correlation was observed between amphetamine and injection drug, with a Pearson correlation of 0.50, followed by the correlations between use of sedatives and number of substances (0.45), and cannabis and number of substances (0.37), respectively. Other correlations were less than 0.3.

In the first set of analyses, each pair of index crime and recidivist crime was investigated for each potential risk factor using Cox regression. Each one of these equations were run for a single covariate or risk factor at a time. Results were presented as hazard ratios (HR) with a 95% CI. Results were considered significant if *p* was less than 0.05. Only variables with significant results in these primary binary analyses, for each pair of index and recidivist crime, were then further entered into a second multivariate Cox regression analysis, controlling for one another, again resulting in HR presenting with a 95% CI.

## 3. Results

### 3.1. Participant Characteristics

Age at the time of inclusion ranged from 18 to 66 years of age and 90% were male. Clients were followed for an average of 2.7 years. Table 1 contains further details on demographic data, lifetime history of psychiatric problems and substance misuse, as well as criminal history.

The univariate analyses testing potential risk factors with regard to index and recidivist crime yielded a number of potential risk factors at the significance level of *p* < 0.05, which were then run in the multivariate analyses. The factors significantly associated with any offences, with violent crime, drug crime and property crime, respectively, are included in Table 2 (for all crimes as index crime), Table 3 for violent crime as index crime, Table 4 for drug crime as index offence, and Table 5 for those with a property crime as index crime. However, for individuals who had committed a violent crime and reoffended violently, only one risk factor was significant: having committed prior property crime (HR: 1.8, 95% CI 1.0–3.1, *p*-value 0.04).

### 3.2. Multivariate Analysis

During the follow up, 68% committed a second offence. Use of cannabis and sedatives were associated with reduced risks of reoffending. Young age, male gender, psychiatric covariates, as well as homelessness and prior criminality, amphetamine use, and injection drug use increased risks of reoffending. Results are displayed in Table 2. However, these risk factors differed when analysing different crimes. 

In this population, 7.1% reoffended violently and this was associated with young age, male gender, alcohol, psychiatric inpatient treatment and prior violent crime (Table 2). Amongst those who had a violent crime as index crime, 12% reoffended violently, and in this group, none of the risk factors above increased the risk of offending. Since only one risk factor from the univariate analysis was significantly associated with violent reoffending, no multivariate analysis could be made. Multivariate analyses for general reoffending are displayed in Table 3. Amongst those who had a drug crime or property crime as index crime, 3.8% and 7%, respectively, reoffended with a violent crime (risk factors are displayed in Table 4 and Table 5, respectively).

Drug crime recidivism rates were 21%. This was associated with heroin, injection drug use, prior drug and property crime, whereas alcohol and young age were associated with reduced recidivism (Table 4). Amongst those with a drug crime as index crime, 34% committed a new drug crime and the risk factors were similar (Table 4).

General recidivism rates to property crime were 19%. Alcohol reduced the risk of reoffending. Young age, heroin, injection drug use, homelessness and prior property crime were risk factors (Table 5). Amongst those with a property crime, 32% committed a new property crime, which, in this group, was associated with a number of substances misused, as well as prior drug and property crime (Table 5).

## 4. Discussion

The results of this study, assessing risk factor of criminal recidivism in a cohort of prisoners with problematic substance use, appear to support the hypothesis that different risk factors, including the use of specific substances, predict different patterns of reoffending. In this population, the use of sedatives and cannabis decreased the risk of overall criminal recidivism. This was interesting, as research on sedatives and cannabis in relation to criminality has been somewhat inconclusive [2,3].

Risk factors for general recidivism were not surprisingly associated with heavier addiction; amphetamine use and injection drug use, as well as psychiatric covariates, homelessness, young age, male gender and prior prosecutions for drug, violent and property crime. What is interesting about this is that many of these risk factors were not significant when analysing each index crime separately. Not only did risk factors vary between different index crimes but also between different categories of criminal recidivism.

Regarding violent crime, this study suggests that alcohol increases the risk of violent reoffending but is of no importance regarding general criminal recidivism in this population. This further highlights the well-known connection between alcohol use and aggression [3]. It is evident that treatment of alcohol addiction is an important strategy in violence prevention.

We did not find any association between amphetamine use and violent crime. Interestingly, a Canadian study on prison clients with SUD concluded that methamphetamine did not increase the risk of returning to the criminal justice system as a result of violent criminal offending, yet increased the risk of self-reported violent crime [23]. Research on amphetamine and violent crime has been inconclusive and further investigation of such a correlation is much needed [2,3]. It cannot be excluded that a longer follow-up would be needed to evaluate if such a correlation exists.

Amongst those with a violent index crime, only a prior prosecution for property crime predicted violent reoffending. Interestingly, in this group of clients with a violent index crime, gender was of no importance, nor was young age, factors commonly associated with violent crime [12]. This is important, since women with substance use problems convicted for violent crime suffer the same risk of reoffending violently as their male counterparts.

It has been argued that early onset substance and alcohol use are important risk factors for violent crime amongst women [44]. A Finnish study examining the characteristics of female violent offenders concluded that women with severe SUD were more frequent to reoffend violently than women with no SUD and that this group of women often had personality disorders and a non-violent criminal history [45]. It is possible that the women in this population who have offended violently suffer from a combination of severe circumstances, including substance use, and may have reached a threshold where they are as likely as men to offend violently. If so, these women are in even greater need of substance use treatment and rehabilitation in general.

Based on the findings of the present paper, crime-preventing efforts in this group of violent offenders with substance use are much needed, since 12% reoffended violently. Apart from substance use, this study confirmed that the generally accepted risk factors for violent recidivism, i.e., psychiatric disease, male gender, young age and prior violent crime, were also risk factors for future violent crime in this population [12].

General recidivism into drug crime was, as suspected, predicted by heroin and injection drug use, as well as prior prosecution for drug and property crime. Old age was an unexpected risk factor for recidivism to drug crime. This is interesting, since young age is generally linked to criminal recidivism. Alcohol lowered the risk of reoffending within drug crime in this population of substance users, which might be a result of its unique status of being legal.

Another interesting result regarding both drug crime and property crime was that gender was not a significant risk factor for recidivism. It has been discussed that women with SUD are more likely to be involved with drug-related crime, but, to date, the common notion is that gender has very little or no effect on drug-related crime [2]. General recidivism to property crime was predicted by almost the same risk factors as drug crime, except for prior prosecution for drug crime (which did not predict property crime) and the addition of homelessness as a risk factor for property crime. This supports the idea that property crime and drug crime are very closely linked. The economic gains of crime in order to sustain an addiction were, in an Australian study, accountable for 45% of criminal acts amongst people with an addiction to heroin [46].

We could not see any relationship between recidivism into property crime and calming substances. High doses of benzodiazepines have, in other studies, been linked to recidivism to property crime and crime in general amongst clients who inject drugs [29,30]. It should be noted that the clients in these studies used benzodiazepines as a supplement to heroin or an injection drug use [29,30]. Furthermore, this study did not take into consideration dosages and did not investigate the effect of benzodiazepines separately, but rather at the effect of all sedatives.

In the analysis of the group sentenced for property crime, 80% committed a recidivist crime within follow-up. Given the relatively limited follow-up time, i.e., an average of 2.7 years, this must be considered a high level of recidivism. In a previous domestic publication, describing a study by the Swedish criminal justice system, 65 percent of prisoners relapsed into crime within a three-year follow up period, i.e., within a follow-up time similar to the time frame assessed in the present study [28]. General criminal recidivism was, in this group, linked to a criminal history of drug and property crime. Overall, it can be concluded that the high rates of recidivism in the present study is due to the fact that included individuals constituted a substance-using sample. It is evident that further crime-preventing efforts must be made amongst clients with substance use disorders, particularly those convicted for a history of drug and property crime. 

Research on drug courts, where clients with problematic substance use involved in non-violent crime are monitored and treated for their substance use, has shown promising results in the US and in Canada [47,48,49]. Notably, drug courts seem to be less effective for younger clients [49], which is interesting since this study considered older age a risk factor for recidivism to drug crime. In addition, it is of importance to conclude that in treatment programs for offenders, treatment retention is likely a key factor in preventing recidivism [50].

In the present study, younger age was not universally a risk factor for recidivism, but for violent crime, being younger increased the risk. Somewhat unexpectedly, older age was associated with a relapse into drug crime. However, despite this, it is likely that attention must be paid to younger clients in samples such as the present one with substance-using clients. In addition, it has been suggested that this group is in need of different crime preventing measures [49]. In the meta-analysis by Bennet et al., on drug use and crime, it is stated that the drug–crime connection seems much stronger for adults than for youths, but that further research on the subject is needed [2]. However, achieving substantial results in young institutions for young delinquents appears to be a challenge; rates of recidivism are high and based on static baseline factors such as age at first conviction [51]. In line with the drug courts implemented for adults in many settings, juvenile drug courts, paying attention to the particular needs of young offenders, have been suggested and examined, although with mixed and modest effects compared to traditional judicial processes. Further research with stronger research designs has been called for [52].

Altogether, given the role of substance use, and substance-specific associations in this regard, it would seem beneficial to include actual substance use disorder in the verdict also in the present setting. In Sweden, formal drug courts are not available, although a regular court may choose to sentence a person to substance use disorder treatment instead of a custodial sentence. However, the treatment referred to may be diverse, and may in many cases be conducted in the context of social services outside of the medical area, and less frequently in a medical, specialized psychiatric setting. The treatment provided is partly based on local tradition and availability and may not be specific to the patient’s specific substance of mental health disorders [53]. A different intervention available for substance use disorders within the context of non-voluntary treatment is the compulsory care act for individuals with either alcohol or drug use disorders, and which include unvoluntary residential treatment and a gradual transfer to out-patient treatment. Such unvoluntary treatment has been described to primarily reach patients with severe substance use disorders, and post-treatment mortality is high, highlighting the large treatment needs in this group [54,55]. Thus, evidence-based treatment efforts, tailored for diagnostic categories and for specific substance types, are clearly needed and need to be implemented in the format of chains of referral from the judiciary system to treatment systems.

### 4.1. Limitations

Information on baseline data is self-reported, which implicates a risk of recall bias. Additionally, diagnostic data describing substance use or other disorders were not available at baseline in the prison setting and, therefore, the significance of the substance use problem was defined as the presence of a primary drug reported (or a combination of primary drugs) in the ASI interview. Here, diagnostic data would have improved the understanding of the baseline substance-related problem. Furthermore, we had no information on any potential change in behaviour between baseline data and potential recidivism. Another limitation is the fact that the collected data are relatively old. According to the overall, national statistics of the criminal justice system [56], a three-year relapse into crime (leading to a new verdict) is reported to have been relatively stable from the year of 2000 until around 2007 (from 36–40 percent), while, thereafter, it decreased to 30 percent in 2011–2012, where the data thereafter have remained stable. Thus, although not studied in scientific analyses but reported in statistical reporting of the relevant authority, relapse rates appear to have decreased in recent years. This, however, is unlikely to change the overall conclusions of the present paper regarding which of the substance-related and other client characteristics affect recidivism. However, criminal recidivism in the present setting and other countries requires further study, also taking potential longitudinal changes into account. 

Examining the number of relapses to criminal offending, as well as criminal acts for which the clients were not sentenced, are two important aspects that the present study could not evaluate. Assessing levels of substance intake over time would also have been valuable. It has, for example, been suggested that high doses of sedatives increase the risk of aggressive behaviour [18]. Furthermore, evaluating substance intake just prior to criminal offending might also have added to the knowledge on substance use and criminal offending.

Another interesting aspect would have been to investigate the impact of anabolic androgenic steroids (AAS) and gambling addiction and their relation to criminal recidivism. AAS have, in some studies, shown to increase the risk of violent behaviour and crime amongst illicit drug users [57]. AAS have also shown to increase aggression amongst users with no other SUD [58]. The ASI version in this study did not contain information on AAS in contrast to the version used today in Sweden [59]. Evaluations of gambling addiction are also not included in the ASI, although a growing new field of research on gambling and crime indicates that gambling addiction is very common in the prison population [60,61,62,63]. A Danish population study from 2016 also concluded that gambling disorders increase the odds of offending in general, as well as in each of the categories of crime dealt with in this study (violent crime, drug crime and property crime) [64].

As mentioned earlier, research on criminal recidivism is complicated, partly due to differences in legislative systems across nations. Additionally, differences in trends of substance use make it hard to compare research internationally. Furthermore, different methods of crime prevention and differences in length of imprisonment make research hard to compare. In addition, the duration of the baseline prison sentence would have also been of interest to assess in the present study, and the lack of this information in the study may also present a limitation.

### 4.2. Strengths

The study is based on a relatively large data set with rather detailed information, not only on substance use but also on other potential risk factors for criminal recidivism. Furthermore, the cohort consists of a group of people for whom there is a great clinical importance to investigate risk factors for criminal recidivism.

## 5. Conclusions

The results of this study are consistent with the hypothesis that different risk factors predict different patterns of reoffending. In summary, risk factors varied greatly depending on index crime and involved areas of substance use disorders, psychiatric problems, age, gender, homelessness and criminal history. Criminal recidivism to property crime and drug crime were predicted by similar risk factors, whereas violent crime had a unique set of risk factors.

Heroin and injection drug use seem to mediate drug and property crime. Alcohol increased the risk of violent reoffending yet had no impact on general criminal recidivism. Many of the commonly accepted risk factors were of no importance when taking into account which index crime the client had committed.

Male gender was quite unexpectedly linked to neither property crime nor drug crime recidivism. Nor was male gender a risk factor for violent recidivism amongst those with a violent index crime. This is important when implicating individualized care in a more efficient way. Identifying crime-specific risk factors should be an important part of improving rehabilitation into society after imprisonment. Interventions to reduce substance use might decrease recidivism rates. Finally, further research on crime-specific and substance-specific recidivism is required to fully understand the relationship between criminal relapse and addiction.

## Figures and Tables

**Table 1:** Characteristics of clients with and without return to the criminal justice system (*n* = 4207). Displaying demographic data, lifetime history of psychiatric problems and substance misuse, as well as criminal history.

Variable	Percentage (%)	Mean
Number of substances misused		1.8 (≤6)
Substance misuse during one year of		
- Alcohol	46	
- Heroin	22	
- Other opioids	15	
- Sedatives	36	
- Cocaine	16	
- Amphetamine	64	
- Cannabis	62	
Injection drug use for one year	53	
Prior episode of psychiatric inpatient treatment	16	
Psychiatric medication at any time	39	
Prior suicide attempts	23	
Homeless 30 days prior to intake to the criminal justice system	22	
Age		33 years (18–66 years)
Male gender	90	
Female gender	9.9	
Self-reported prior prosecution for:		
- Drug crime	80	
- Violent crime	68	
- Property crime	78	

**Table 2:** Variables associated with return to the criminal justice system including All Offences as index crime. Multivariate Cox regression analysis.

Risk Factors	Recidivist Crime											
	All Offences			Violent Crime			Drug Crime			Property Crime		
	HR	95% CI	*p*	HR	95% CI	*p*	HR	95% CI	*p*	HR	95% CI	*p*
Number of substances	1.0	0.99–1.1	0.2				1.0	0.98–1.1	0.2	1.1	1.0–1.2	>0.05
Alcohol				* 1.5	1.2–2.0	<0.01	* 0.74	0.64–0.87	<0.01	* 0.81	0.69–0.95	<0.01
Heroin	1.09	0.99–1.2	0.1				* 1.3	1.1–1.5	<0.01	* 1.3	1.1–1.6	<0.01
Other Opioids										0.88	0.71–1.1	0.2
Sedatives	* 0.91	0.82–1.0	0.04							1.0	0.87–1.2	0.7
Amphetamine	* 1.1	1.0–1.3	0.01				1.1	0.95–1.4	0.2	1.2	0.95–1.4	0.1
Cannabis	* 0.90	0.82–0.99	0.02							0.98	0.82–1.2	0.8
Injection	* 1.4	1.3–1.6	<0.01				* 1.6	1.3–1.9	<0.01	* 1.4	1.1–1.7	<0.01
Psychiatric Inpatient Treatment	* 1.1	1.0–1.3	0.02	* 1.5	1.1–2.0	<0.01						
Psychiatric Medication	* 1.2	1.1–1.1	0.01	1.2	0.97–1.6	0.09				1.1	0.95–1.3	0.2
Suicide Attempt	0.95	0.86–1.0	0.3									
Homelessness	* 1.2	1.1–1.3	<0.01				1.0	0.86–1.2	0.8	* 1.4	1.1–1.6	<0.01
Age	* 0.98	0.98–0.99	<0.01	* 0.98	0.97–1.0	<0.01	* 1.0	1.0–1.0	<0.01	* 0.96	0.95–0.97	<0.01
Male Gender	* 1.4	1.2–1.6	<0.01	* 3.2	1.6–6.2	<0.01						
Prior Drug Crime	* 1.3	1.1–1.4	<0.01	0.90	0.68–1.2	0.4	* 2.5	1.9–3.3	<0.01	1.1	0.89–1.4	0.4
Prior Property crime	* 1.2	1.1–1.3	0.001	1.2	0.93–1.7	0.2	* 1.3	1.0–1.5	0.02	* 3.1	2.3–4.1	<0.01
Prior Violent Crime	* 1.8	1.6–2.0	<0.01	* 2.8	2.0–4.1	<0.01	0.92	0.80–1.1	0.3	1.2	0.97–1.4	0.1

* Hazard ratio is significant at the 0.05 level (2-tailed). Note: Hazard ratios are rounded off and displayed with two significant figures. *p*-values are displayed as one significant figure.

**Table 3:** Variables associated with return to the criminal justice system including Violent Crime as index crime. Multivariate Cox regression analysis.

Risk Factors	Recidivist Crime					
	All Offences			Violent Crime		
	HR	95% CI	*p*	HR	95% CI	*p*
Number of Substances	0.99	0.89–1.1	0.8			
Alcohol	0.93	0.74–1.2	0.6			
Heroin	1.3	0.99–21.8	0.06			
Sedatives	1.0	0.80–1.3	0.8			
Cocaine	1.2	0.85–1.6	0.4			
Amphetamine	1.1	0.84–1.4	0.5			
Cannabis	0.92	0.72–1.2	0.5			
Injection	1.3	0.98–1.6	0.07			
Psychiatric Inpatient Treatment	1.2	0.93–1.6	0.2			
Homelessness	1.2	0.95–1.6	0.1			
Age	* 0.98	0.96–0.99	<0.01			
Male Gender	* 1.8	1.1–3.0	0.02			
Prior Drug Crime	* 1.3	1.0–1.7	0.04			
Prior Property crime	1.2	0.94–1.6	0.1	* 1.8	1.0–3.1	0.04

* Hazard ratio is significant at the 0.05 level (2-tailed). Note: Hazard ratios are rounded off and displayed with two significant figures. *p*-values are displayed as one significant figure.

**Table 4:** Variables associated with return to the criminal justice system including Drug Crime as index crime. Multivariate Cox regression analysis.

Risk Factors	Recidivist Crime								
	All Offences			Violent Crime			Drug Crime		
	HR	95% CI	*p*	HR	95%CI	*p*	HR	95% CI	*p*
Number of Substances	1.0	0.97–1.2	0.2				1.1	0.97–1.2	0.2
Heroin	1.1	0.95–1.4	0.2				* 1.3	1.0–1.6	0.04
Sedatives	0.94	0.78–1.1	0.5				1.0	0.79–1.3	0.9
Amphetamine	1.2	0.96–1.4	0.1				1.3	0.99–1.6	0.07
Cannabis				1.9	0.95–3.6	0.07			
Injection	* 1.5	1.3–1.8	0.00				* 1.4	1.1–1.9	0.004
Psychiatric Inpatient Treatment	1.2	0.93–1.5	0.2						
Psychiatric Medication	1.1	0.95–1.3	0.2						
Suicide Attempt	0.85	0.69–1.0	0.1						
Homelessness	1.1	0.94–1.4	0.2				0.99	0.76–1.3	1.0
Age	1.0	0.99–1.0	0.6	* 0.95	0.92–0.98	<0.01	* 1.0	1.0–1.0	0.001
Prior Property Crime	* 1.7	1.4–2.1	<0.01				* 1.4	1.1–1.8	0.007
Prior Violent Crime	* 1.4	1.2–1.7	<0.01	* 3.1	1.7–5.6	<0.01			

* Hazard ratio is significant at the 0.05 level (2-tailed). Note: Hazard ratios are rounded off and displayed with two significant figures. *p* values are displayed as one significant figure.

**Table 5:** Variables associated with return to the criminal justice system including Property Crime as index crime. Multivariate Cox regression analysis.

Risk Factors	Recidivist Crime								
	All Offences			Violent Crime			Property Crime		
	HR	95% CI	*p*	HR	95% CI	*p*	HR	95% CI	*p*
Number of Substances							* 1.1	1.0–1.2	0.05
Alcohol							* 0.76	0.61–0.95	0.02
Heroin	1.0	0.89–1.2	0.6				1.2	0.95–1.6	0.1
Other Opioids							1.1	0.81–1.4	0.62
Sedatives							1.0	0.79–1.3	1
Cocaine	0.93	0.78–1.2	0.5						
Amphetamine	0.98	0.83–1.2	0.9				1.1	0.86–1.5	0.4
Injection	1.2	0.98–1.4	0.09				0.93	0.72–1.2	0.6
Psychiatric Inpatient Treatment	1.1	0.92–1.3	0.3	1.4	0.82–2.4	0.2			
Psychiatric Medication	1.2	1.0–1.3	(>)0.05	* 1.6	1.0–2.5	0.04			
Homelessness	* 1.2	1.0–1.4	0.02				1.2	0.91–1.5	0.2
Age	1.0	0.99–1.0	0.9						
Gender				2.6	0.81–8.4	0.1			
Prior Drug Crime	* 1.5	1.3–1.8	<0.01				* 1.4	1.0–1.9	0.02
Prior Property crime	* 1.6	1.2–2.1	<0.01				* 2.5	1.5–4.2	<0.01
Prior Violent Crime				1.9	0.96–3.9	0.06	0.85	0.67–1.1	0.2

* Hazard ratio is significant at the 0.05 level (2-tailed). Note: Hazard ratios are rounded off and displayed with two significant figures. *p*-values are displayed as one significant figure.

## Data Availability

The data used to support the findings of this study were provided by the Swedish Criminal Records Registry and the Swedish National Board of Health and Welfare under license. Anonymized data can be made freely available only after application to these authorities. In order to make this request an approval from the ethcis review authority of Sweden will be necessary.
